# Non-classical animal models for studying adrenal diseases: advantages, limitations, and implications for research

**DOI:** 10.1186/s42826-024-00212-8

**Published:** 2024-06-19

**Authors:** Alina Bilyalova, Airat Bilyalov, Nikita Filatov, Elena Shagimardanova, Andrey Kiyasov, Maria Vorontsova, Oleg Gusev

**Affiliations:** 1 Institute of fundamental medicine and biology, Kazan Federal University, Kazan, 420008 Russia; 2 Loginov Moscow Clinical Scientific Center, Moscow, 111123 Russia; 3 Lomonosov Moscow State University, Moscow, 119991 Russia; 4 Life Improvement by Future Technologies (LIFT) Center, Moscow, 121205 Russia; 5 Intractable Disease Research Center, Graduate School of Medicine, Juntendo University, Tokyo, 113-8421 Japan; 6 Endocrinology Research Center, Moscow, 117292 Russia

**Keywords:** Animal models, Steroidogenesis, Adrenal gland, CAH, spiny mouse, golden hamster

## Abstract

The study of adrenal disorders is a key component of scientific research, driven by the complex innervation, unique structure, and essential functions of the adrenal glands. This review explores the use of non-traditional animal models for studying congenital adrenal hyperplasia. It highlights the advantages, limitations, and relevance of these models, including domestic ferrets, dogs, guinea pigs, golden hamsters, pigs, and spiny mice. We provide a detailed analysis of the histological structure, steroidogenesis pathways, and genetic characteristics of these animal models. The morphological and functional similarities between the adrenal glands of spiny mice and humans highlight their potential as an important avenue for future research.

## Background

The adrenal glands have always been a subject of scientific interest due to their heterogeneous structure, the variety of hormones that adrenal glands synthesise, complex innervation, and a multitude of physiological functions. They were first mentioned by Bartolomeo Eustachi, who described the adrenal glands as “glandulae quae renibus presentative” (glands located near the kidneys) in his book Opuscola Anatomica, published in 1564 [1]. Up until the mid-19th century, their study was limited to certain anatomical investigations, until Thomas Addison in 1855 discovered that their dysfunction leads to a clinical syndrome, now named after him [1]. However, despite the substantial amount of research and data accumulated on adrenal glands, this organ remains a subject of scientific interest, especially in the context of the search for new methods of diagnosis and treatment of some adrenal diseases.

For example, one form of primary adrenal insufficiency is congenital adrenal hyperplasia (CAH), a group of autosomal recessive disorders with an overall prevalence of 1: 9498 live births in world [2]. Pathogenesis is based on defects in enzymes or transport proteins involved in the biosynthesis of steroid hormones in the adrenal cortex [3]. Insufficiency arises from mutations in genes that encode enzymes of various steps in the biosynthesis of steroid hormones, leading primarily to decreased cortisol synthesis and hyperproduction of adrenocorticotropic hormone (ACTH) due to altered negative feedback loops. As a result, adrenal cortex hyperplasia develops and the target hormone and precursor accumulate above the enzymatic block [3, 4].

Currently, the predominant therapeutic approach to address CAH involves the administration of lifelong replacement hormonal therapy. This therapy serves the purpose of mitigating endogenous hormone deficiency while simultaneously suppressing the secretion of corticotropin-releasing hormone and ACTH, thus restoring the perturbed feedback system through exogenously supplied hormonal agents adrenocorticotropic hormone [4]. However, despite this, this therapeutic modality has no limitations. For example, the administration of hormones based on exogenous tablets poses challenges in the context of infants and very young children. Furthermore, the therapeutic regimen fails to faithfully recapitulate the intricate physiological diurnal rhythm of hormone secretion or response to stressors. The potential stunting of growth in paediatric patients subjected to routine glucocorticoid therapy is of note. The process of determining the optimal dose requires extensive and sustained adjustments, and patient adherence may not always be reflected in the intended therapeutic outcome. Rehabilitation of disrupted negative feedback mechanisms may be suboptimal in certain cases, examples, highlighting a pertinent issue [5]. Furthermore, prolonged administration of glucocorticoids, especially at supraphysiological levels, is associated with the emergence of adverse effects that include obesity, insulin resistance, sarcopenia, dermal atrophy, and other sequelae [6, 7].

In recent years, efforts have been made to develop new glucocorticoid delivery systems or alternative therapeutic approaches that do not involve glucocorticoids. However, the question of fully correcting the symptoms of adrenal insufficiency remains relevant [8]. A radically new approach to treating this condition is based on genetic and cellular technologies. Currently, there is active development in genome editing tools delivery systems, various approaches to gene cellular therapy, gene cellular, and protocols for the culture of functional adrenal cortex cells in vitro [9].

Advancement of genetic and cellular treatment techniques requires preliminary investigations conducted in animal models, often using mice and rats. However, an investigation by Marjut Pihlajoki et al. underscores the substantial morphological and functional disparities between the adrenal glands of these animals and those of humans [10]. There is no zona reticularis (ZR) in the adrenal gland of mice and rats and therefore there is no androgen production [10]. In addition, in rats, there is an undifferentiated zone located in the cortex, which is situated between the zona glomerulosa (ZG) and zona fasciculata (ZF) [11]. It has been suggested that stem and/or progenitor cells expressing steroidogenic factor-1, which has been confirmed by immunohistochemistry study [11]. In contrast to the ZR, young mice exhibit an X-zone that disappears upon reaching sexual maturity in males and during pregnancy or within 3–7 months postpartum in the absence of pregnancy in females [10, 12]. This review will describe alternative animal models that could be used to study CAH.

## Main text

### The structure of the human adrenal glands and steroidogenesis

To foster a more comprehensive grasp of the distinctive strengths and constraints inherent to each model, a brief elucidation is warranted concerning the configuration of human adrenal glands and the process of steroidogenesis before embarking on the discourse surrounding alternative animal models for the examination of CAH.

The adrenal glands are paired parenchymal organs of the endocrine system, consisting of the adrenal medulla and adrenal cortex, each of which performs specific functions and has different origins [13].

The adrenal medulla develops from neural crest cells and is located within the ZR of the adrenal cortex and is made up of three types of cells: chromaffin cells (pheochromocytes), ganglion cells, and supporting cells [14–16]. Chromaffin cells are the predominant cell type in the adrenal medulla, forming clusters of cells that synthesise catecholamines (norepinephrine and epinephrine). Supporting cells are located around these clusters, while ganglion cells are dispersed individually or form clusters between chromaffin cells or near nerve fibres [17, 18].

The adrenal cortex develops from the embryonic mesoderm and synthesises steroid hormones. The adrenal cortex is encased in a fibrous capsule, also derived from the mesoderm, which serves protective and supportive functions and acts as a reservoir for stem cells from the adrenal cortex [19, 20]. In the context of humans, the adrenal cortex manifests three discernible zones characterised by different morphological and functional attributes: ZG, ZF, and ZR [20].

The outermost layer, known as ZG, represents approximately 5–15% of the total cortical volume. It acts as the primary site for the biosynthesis of mineralocorticoids, predominantly aldosterone. This vital hormone plays a crucial role in the meticulous regulation of potassium and sodium levels in the bloodstream [21].

Below the ZG lies the ZF, which occupies up to 75% of the total volume of the cortex and synthesises glucocorticoids, primarily cortisol, together with a smaller number of androgens [22]. Glucocorticoids play a crucial role in the body’s stress response, influencing metabolism, particularly carbohydrate metabolism, and to a lesser extent electrolyte balance due to some mineralocorticoid activity [23–26].

The innermost layer of the cortex, recognised as ZR, represents approximately 10–20% of the total cortical volume. Within this region, the synthesis of steroid hormones occurs, encompassing mildly active androgens such as DHEA, DHEA-S, androstenedione, androstenediol, 11β-hydroxyandrostenedione, more biologically active androgens like 11β-hydroxytestosterone and testosterone, in addition to a small amount of glucocorticoids [13, 22, 27].

**Fig. 1:**
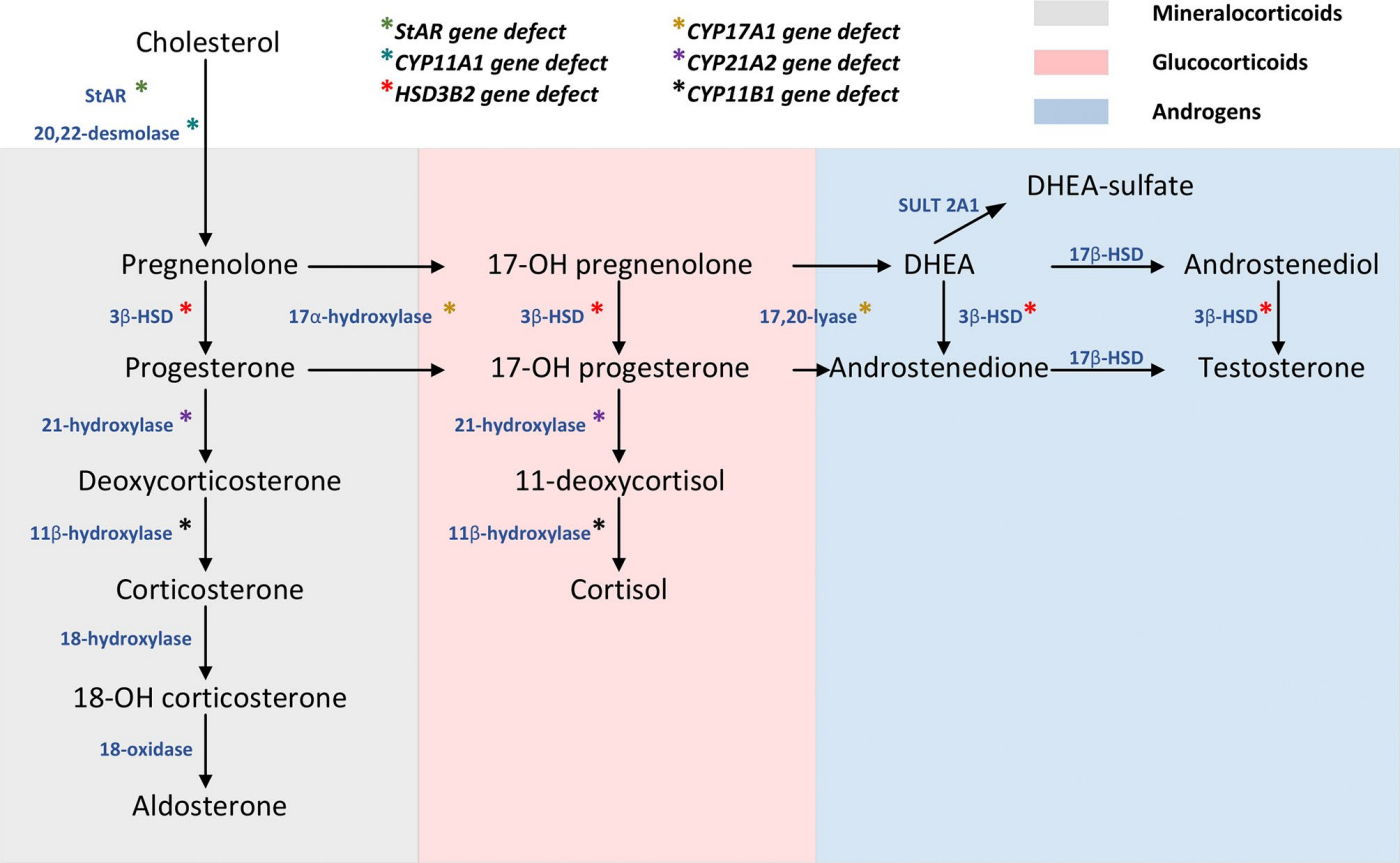
Scheme of steroidogenesis and CAH types. StAR -steroidogenic acute regulatory protein; 3β-HSD − 3 β -hydroxysteroid dehydrogenase; 17β -HSD − 17β -hydroxysteroid dehydrogenase; DHEA - dehydroepiandrosterone; SULT 2A1 - sulfotransferase

The process of steroid hormone biosynthesis in the adrenal cortex begins with cholesterol and is subject to regulation by the adrenocorticotropic hormone (ACTH) secreted by the anterior pituitary (Fig. 1). Cholesterol can be obtained from dietary sources, particularly those of animal origin. Furthermore, within the adrenal glands and ovaries, cholesterol can be synthesised de novo from acetate. Furthermore, a proportion of cholesterol is manufactured within the liver, predominantly in the form of low-density lipoproteins [28, 29].

The beginning of steroid hormone synthesis is induced by the acute steroidogenic regulatory protein (StAR), which facilitates the translocation of cholesterol from the cytoplasm to the outer and, subsequently, inner mitochondrial membranes [30, 31]. Within mitochondria, cholesterol undergoes the conversion to pregnenolone under the catalytic influence of the enzyme 20,22-desmolase (encoded by the *CYP11A1* gene). This transformation involves a series of successive hydroxylation reactions, culminating in the cleavage of the cholesterol side chain. Once pregnenolone is generated, which serves as the precursor of all steroid hormones that include mineralocorticoids, glucocorticoids, androgens, estrogens, and progesterone, it is translocated from the mitochondria to the endoplasmic reticulum [32].

Pregnenolone undergoes a 17α-hydroxylation reaction catalysed by the 17-hydroxylase enzyme 17α-hydroxylase to form 17-hydroxypregnenolone, which can then be converted to progesterone by the enzyme 3β-hydroxysteroid dehydrogenase (3β-HSD). The 17α-hydroxylase catalyses carbon atom hydroxylation at position 17, while 17,20-lyase catalyses side chain cleavage at positions 17 and 20, leading to the formation of 19-carbon precursors of sex steroids. The enzyme 3β-HSD converts pregnenolone to progesterone and DHEA to androstenedione, and the enzyme 17β-hydroxysteroid dehydrogenase (17β-HSD) is involved in the formation of testosterone from androstenedione [33]. In the absence of 17-hydroxylase activity (for example, in the ZG of the adrenal cortex), pregnenolone is converted to mineralocorticoids. If 17α-hydroxylase activity is present in the absence of 17,20-lyase activity (for example, in the ZF of the adrenal cortex), pregnenolone is converted to cortisol through the glucocorticoid pathway [33].

### Classification and etiology of congenital adrenal hyperplasia (CAH)

Before delving into the discussion of non-classical animal models, it is worth mentioning a few words about the classification and clinical manifestations of CAH. Currently, seven clinical genetic variants of CAH are identified (Fig. 1) [34]:

1. Lipoid hyperplasia of the adrenal cortex (*StAR* gene defect).
2. Lipoid hyperplasia of the adrenal cortex − 20,22-desmolase deficiency (*CYP11A1* gene defect).
3. 17α-hydroxylase/17,20-lyase deficiency (*CYP17A1* gene defect).
4. 3β-hydroxysteroid dehydrogenase deficiency (*HSD3B2* gene defect).
5. 21-hydroxylase deficiency (*CYP21A2* gene defect).
6. 11β-hydroxylase deficiency (*CYP11B1* gene defect).
7. Oxidoreductase deficiency (*POR* gene defect).

Lipoid hyperplasia of the adrenal cortex develops due to a defect in the *StAR* gene located on the short arm of chromosome 8 (8p11.2) or a defect in the *CYP11A1* gene located on chromosome 15 (15q23-24) [35–37]. Deficiency of the StAR protein, as well as 20,22-desmolase, leads to the development of a severe form of CAH characterised by the absence of synthesis of all classes of steroids in both the adrenal glands and the gonads (ovaries, testes) [38, 39].

CAH caused by defects in the *CYP17A1* gene, located on chromosome 10 (10q24.3), is characterised by a deficiency of both 17α-hydroxylase and 17,20-lyase [40]. The lack of these enzymes leads to a deficiency of glucocorticoids and androgens. Deficiency of 17α-hydroxylase disrupts cortisol synthesis, leading to ACTH hyperproduction and activation of aldosterone precursor synthesis, while deficiency of 17,20-lyase leads to impaired androgen synthesis in adrenal glands and gonads [41, 42]. Mutations in the *CYP17A1* gene are relatively rare, with several mutations reported that cause complete or combined deficiency of the 17-hydroxylase / 17,20-lyase enzyme or isolated 17,20-lyase deficiency [43–47].

The deficiency of 3β-hydroxysteroid dehydrogenase type 2 3-hydroxysteroid dehydrogenase (HSD3B2) is extremely rare and is caused by mutations in the *HSD3B2* gene located on chromosome 1 (1p12) [47, 48]. It is characterised by impaired synthesis of all classes of steroid hormones in the gonads and adrenal glands [48–51].

The most common form of CAH, accounting for more than 95% of cases, is due to a deficiency of the enzyme 21-hydroxylase, resulting from mutations in the *CYP21A2* gene located on chromosome 6 (6p21.33) [52, 53]. The *CYP21A2* gene is located in tandem with a highly homologous pseudogene, *CYP21A1P.* Unequal crossover between these genes during meiosis leads to deletions and the formation of non-functional chimeric genes [54]. The 21-hydroxylase enzyme belongs to the cytochrome P450 monooxygenase family, is located in the cisterns of the endoplasmic reticulum and is involved in the conversion of progesterone and 17-hydroxyprogesterone to 11-deoxycorti-costerone and 11-deoxycortisol, respectively [55–58]. Depending on the amount of the active 21-hydroxylase enzyme, two clinical forms of 21-hydroxylase deficiency are distinguished: classical and non-classical. The classical form, observed in approximately 75% of cases, is characterised by low enzyme activity, resulting in a deficiency of not only cortisol, but also aldosterone and excess testosterone production due to the diversion of precursor hormones toward androgen production. The non-classical form develops with preserved enzyme activity at 20–50%, characterised by a less significant reduction in cortisol production and excessive androgen synthesis after puberty [59, 60].

Deficiency of 11β-hydroxylase arises from a mutation in the *CYP11B1* gene located on chromosome 8q21 [61]. This enzyme catalyses the addition of hydroxyl groups, converting 11-deoxycortisol and 11-deoxycorticosterone into cortisol and corticosterone, respectively [62]. Deficiency of 11β-hydroxylase disrupts cortisol synthesis, which, through a negative feedback mechanism, stimulates ACTH synthesis and leads to the formation of cortisol and aldosterone precursors, as well as androgens [63]. Furthermore, 11-deoxycorticosterone has mineralocorticoid activity and its accumulation leads to the development of arterial hypertension, sometimes with a malignant course that cannot be alleviated with medication [64].

P450 oxidoreductase (POR) is a relatively rare form of CAH, first described in 2004 [65]. This form of CAH is caused by mutations in the *POR* gene, located on chromosome 7 (7q11.2), responsible for the formation of the enzyme P450 oxidoreductase. This enzyme is necessary to provide oxygen molecules to all microsomal enzymes in the cytochrome P450 family, including the previously mentioned CYP17A1 and CYP21A2 [66]. Patients with POR deficiency exhibit a combined partial deficiency of 17α-hydroxylase and 21-hydroxylase deficiency, which clinically often manifests itself as moderately reduced levels of glucocorticoid and mineralocorticoid, as well as elevated androgen concentrations in the blood [67].

According to the literature, CAH is rare in animals. One case of CAH has been described in a cat [66]. A mutation in a gene similar to 11β-hydroxylase is associated with clinical manifestations such as polyuria, polydipsia, and increased blood pressure [68].

### Non-classical emerging animal models

**Fig. 2:**
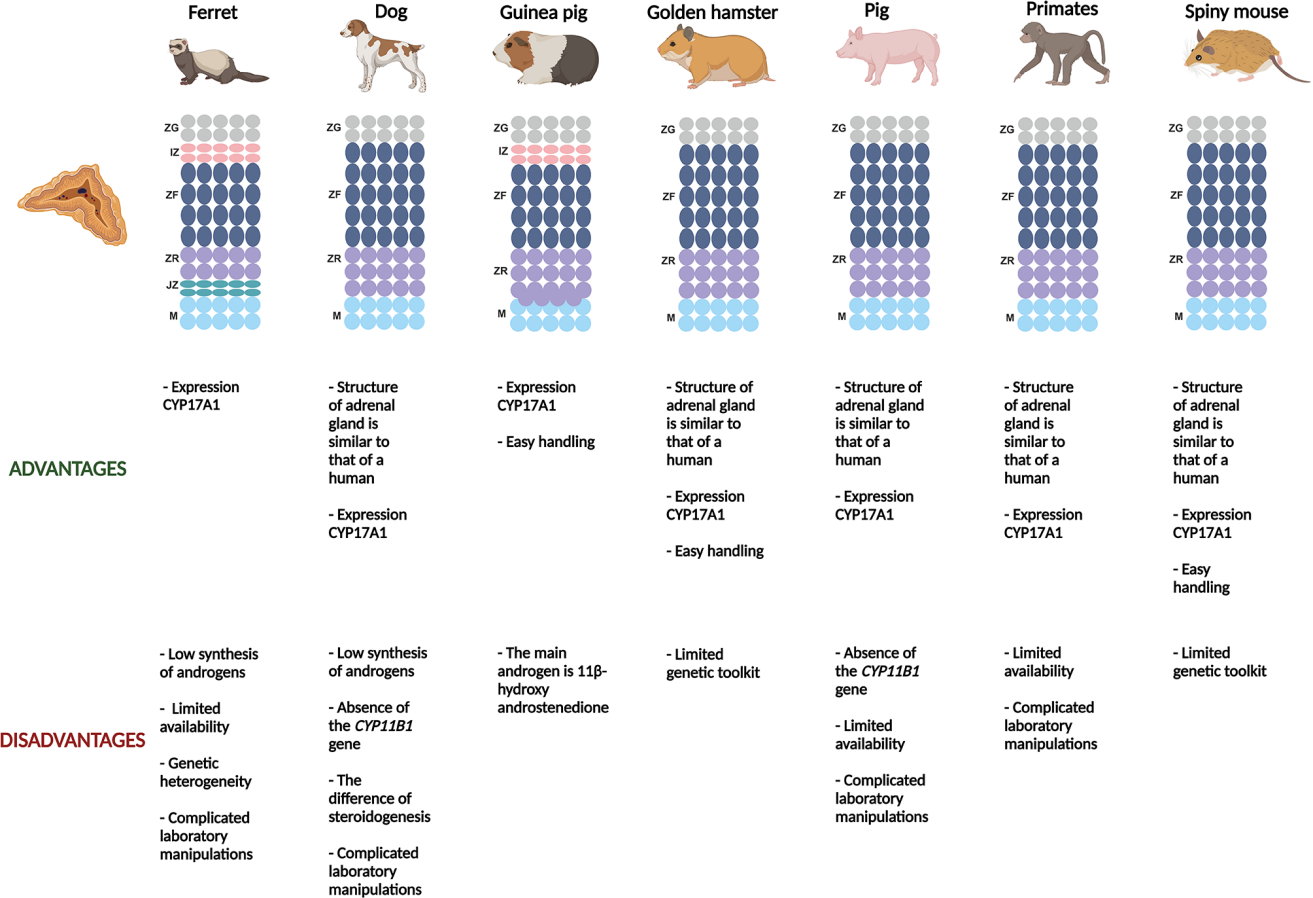
Comparative characteristics of non-classical animal models. ZG – zona glomerulosa; IZ – intermediate zone; ZF – zona fasciculata; ZR – zona reticularis; JZ - juxtamedullary zone; M – medulla

Nonclassical animal models that have the potential to be studied include ferrets, dogs, guinea pigs, golden hamsters, pigs, primates, and spiny mice (Fig. 2).

#### Domestic ferret or ferret

According to the work of Holmes, RL, who first described the structure of adrenal glands in domestic ferrets, their adrenal glands are encapsulated in a thick connective tissue capsule, under which two well-distinguishable parts are identified: the cortex and medulla. Three main zones characteristic of the human adrenal cortex, ZG, ZF, and ZR, are easily distinguishable from each other [69]. Additionally, there are other structures that are not observed in the human adrenal glands. For example, between the ZG and the ZF, an irregularly shaped narrow strip is identified that forms an intermediate zone. At the cellular level, this structure is made up of small cells of irregular shaped with centrally located nuclei. Furthermore, between the ZR and the medulla, a juxtamedullary zone is present [69]. Similarly to human physiology, mineralocorticoids are synthesised in the ZG, glucocorticoids, including cortisol in the ZF, and androgens such as estradiol, 17-hydroxyprogesterone, and androstenedione in the ZR [70].

Within the medulla, prominent chromaffin cells of considerable size can be discerned, characterised by a centrally positioned spherical nucleus and a pale cytoplasm replete with granular elements. Furthermore, clusters of ganglion cells manifest their presence, occupying positions within the confines of the medulla or at the junction that delimits the cortex and medulla [71].

An advantage of domestic ferrets as a model for studying adrenal diseases is their expression of the *CYP17A1* gene, which is responsible for the synthesis of enzymes that catalyse both the 17α-hydroxylation reaction necessary for cortisol production and the 17,20-lyase reaction necessary for androgen synthesis [71, 72]. Cells in ZF and ZR possess 17α-hydroxylase activity, suggesting that cortisol is the main glucocorticoid in this species, similar to humans [73, 74]. It should be noted that androgen synthesis in the adrenal glands of domestic ferrets is limited [75]. This is due to restricted synthesis of the cytochrome B5 enzyme (Cyt-b5), an allosteric enzyme that regulates the activity of 17,20-lyase, which is necessary for androgen synthesis in the adrenal glands [72].

In addition, domestic ferrets serve as a valuable experimental model for a comprehensive investigation of the intricate mechanisms that underlie the process of tumorigenesis within the adrenal cortex. Compared to specific murine species, ferrets subjected to gonadectomy exhibit the emergence and progression of neoplastic growths within the confines of the adrenal cortex, as documented in the study by reference [73]. It is of particular significance to emphasise that cellular entities that originate within the boundaries of the adrenal cortex during the course of this pathological progression demonstrate a predilection for the secretion of androgenic hormones, in contrast to the synthesis and release of mineralo- and glucocorticoids [75]. This biosynthetic activity centred on androgen production within cortical tissue precipitates a physiological state characterised and identified as adrenal-associated endocrinopathy. The clinical manifestations that follow encompass bilateral symmetric alopecia and hypertrophy of the external genitalia, as elucidated in scholarly discourse [76]. Confirmation and validation of this diagnostic categorisation is established through the discernible elevation of circulating concentrations of 17α-hydroxyprogesterone, androstenedione, DHEA-S, or estradiol within the plasmatic environment, as expounded in the publications denoted by references [75, 76].

However, despite the structural parallels that exist between the adrenal glands of domestic ferrets and humans, coupled with the shared expression of the *CYP17A1* gene and the subsequent androgen biosynthetic processes, certain limitations persist that curtail the extensive applicability of these creatures as a suitable experimental paradigm. Among the shortcomings inherent in this model are the challenges associated with the purchase of an adequate cohort of animals, the inherent genetic heterogeneity prevalent within the population, distinctive behavioural attributes, and the imposition of more intricate requirements related to laboratory housing conditions, in stark contrast to comparatively well-established and available model organisms such as rats and mice, as extensively elaborated in the scholarly discourse presented by references [77, 78].

#### Dogs

Dogs can also be considered emerging models for studying adrenal diseases. In the adrenal cortex of dogs, similar to that of humans, three zones are distinguished: ZG, ZF, and ZR [79]. Like other mammals, the ZG regulates sodium and potassium levels in blood plasma, while the ZF and ZR together function as the main source of glucocorticoid secretion [13]. Furthermore, androgens such as DHEA and androstenedione are produced in the ZR [80].

Given the commonality of the end products of adrenal cortical synthesis, specifically aldosterone and cortisol, shared between humans and dogs, the assumption was established that the steroidogenesis processes would exhibit parallelism in both species. In recent literature, the sequence of adrenal cortical steroidogenesis in dogs closely parallels the established human steroidogenesis pathway [81].

In humans, the final stages of aldosterone and cortisol synthesis, as well as the final stages of aldosterone and corticosterone synthesis in rats and mice, are catalysed by two different related enzymes: aldosterone synthase and 11β-hydroxylase, which are encoded by the genes *CYP11B2* and *CYP11B1*, respectively [82, 83]. However, according to the NCBI database, the dog genome contains *CYP11B2* (NC_006595.3) but not *CYP11B1*.

Sanders et al. show that there is a significant gap in the canine genome near the known *CYP11B2* gene sequence and that the *CYP11B1* gene is absent in dogs, which should have been involved in steroidogenesis [84]. Sanders et al. also suggested an alternative scheme of steroidogenesis in dogs without the use of aldosterone synthase [85]. Similar to ferrets, androgen synthesis under physiological conditions is limited in dogs [75, 85]. The reasons for this phenomenon are unknown [86].

Despite the identical structure of the adrenal cortex in dogs and humans, as well as the same end products of synthesis, there are differences in the catalysts of the reaction, one notable example being the absence of aldosterone synthase [84]. Additionally, due to their size and storage conditions, dogs are less commonly used in laboratory research compared to smaller animals with simpler housing conditions, making them a questionable model for studying human adrenal diseases.

#### Guinea pigs

Guinea pigs, classified as domesticated rodents that fall under the genus Cavia and the Caviidae family, are a laudable model for laboratory animal studies due to their body mass, dimensions, ease of manipulation and care prerequisites [87, 88].

The adrenal glands of guinea pigs are covered by a connective tissue capsule, under which the cortex and medulla are distinguished. According to the literature, the adrenal cortex histologically reveals three zones: the ZG, the ZF and the ZR [89, 90]. However, according to Sheikhian A. and others, an intermediate zone is identified between the ZG and the ZF, composed of a thin layer of small irregularly shaped cells with dark stained nuclei. Furthermore, the cortex extends deep into the medulla, revealing structures reminiscent of invaginations [91]. In the study by Lafi A. and colleagues, the ZF of the adrenal cortex is composed of two morphologically distinguishable segments: the outer section comprises polygonal cells organised in parallel bundles, while the inner segment is composed of cells arranged in irregular cords, interspersed with sinuses [89]. Histologically, the medulla contains chromaffin and ganglion cells [89–91].

Immunohistochemical staining of guinea pig adrenal glands using antibodies against three enzymes revealed that the expression of 3β-HSD, 20,22-desmolase is present in all zones of the adrenal cortex, while the expression of 17α-hydroxylase is observed only in the ZF and ZR. The staining was more intense in the outer layer of the ZF and gradually decreased toward the inner layers of the ZF and ZR [92]. The results indicate that the 17-hydroxylase enzyme 17α-hydroxylase is synthesised in these animals, resulting in the formation of cortisol and androgens [92, 93]. It should be noted that steroidogenesis in the adrenal glands of guinea pigs occurs differently than in humans [94]. For example, androgens DHEA and DHEA-S are not detected in guinea pig blood, but another C19 steroid, 11β-hydroxyandrostenedione, is present [95–97].

Despite their near-ideal status as laboratory animals, the configuration of their adrenal glands and variations in the synthesis of specific steroid hormones hinder the use of guinea pigs as an optimal model for investigating adrenal diseases.

#### Cricetidae

The Syrian hamster, known colloquially as the golden hamster, belongs to the rodent family Cricetidae and is one of the most popular species among domesticated animals [98].

Researchers have been interested in the adrenal glands of these animals since the mid-20th century, studying not only their histological structure, but also determining the spectrum of hormones they produce [99–103].

Similarly, for most mammals, the adrenal glands of the Syrian hamster are enveloped by a thin connective tissue capsule, beneath which the cortex and medulla are discernible [102]. The adrenal cortex can be divided into three zones: ZG, ZF, and ZR [91, 101]. In particular, these animals synthesise the enzyme 17α-hydroxylase, 3β-HSD, which participates in the formation of androgens and cortisol [104–106]. The medulla is made up of chromaffin cells [107].

The benefits of this model include its similarity to human adrenal glands in terms of both morphology and function, as well as its relatively simple laboratory maintenance requirements.

Recent study has uncovered significant heterogeneity among several members of the Cricetidae family, directly linked to their genetic characteristics. In particular, a new cortical region, zona inaudita, was identified in the Oldfield mouse (*Peromyscus polionotus*), which expresses the enzyme AKR1C18. This enzyme converts progesterone into 20α-hydroxyprogesterone [108]. We expect this discovery to continue attracting the attention of researchers in the field in the near future, as it opens up new possibilities for understanding the complex genetic underpinnings of these animals.

#### Pigs

Large animals such as pigs are considered nearly ideal animals for modelling various adrenal gland diseases due to their anatomical and physiological similarity to the human paired organ [109–111]. These model animals offer opportunities for the detection of biomarkers during pathological conditions and the study of new drugs [112, 113].

Similarly to humans, the adrenal cortex of pigs consists of three morphological layers: the ZG, the ZF, and the ZR [114]. The end products of steroidogenesis are identical to those of humans.

Twinkle Vohra’s research suggests that pigs could be used as a suitable model for studying primary hyperaldosteronism, due to their similarities with humans [115]. However, rodents lack certain genetic expressions, such as *KCNJ5*, which limits their ability to mimic this condition [115]. On the other hand, pigs exhibit comparable potassium channel gene profiles, overcoming this obstacle.

A limitation of using pigs as animal models to study human adrenal gland diseases is the expression of only one enzyme, CYP11B1, in the adrenal cortex, while humans also have CYP11B2. Additionally, their larger size and complex housing requirements can be considered disadvantages [116].

#### Primates

Primates are important laboratory animals for biomedical, pharmacological and toxicological research. Several taxonomic groups of primates are used in research, including prosimians (e.g. lorises and lemurs), New World monkeys (e.g. marmosets and capuchins), Old World monkeys (e.g. macaques and baboons) and apes (e.g. gibbons, gorillas, orangutans and chimpanzees) and some of them have their own special features [117]. The adrenal glands are very similar to the human adrenal glands: they also have 3 different zones in the cortex and produce androgens, but there are exceptions [117].

A study by Toru Tachibana et al. [118] compared two species of New World monkeys: Aotus spp. (night monkeys) and Saimiri spp. (squirrel monkeys). Both species have a similar adrenal structure, but the adrenal cortex of Aotus spp. showed a significant enlargement of the ZF, and the fasciculata cells showed a remarkable accumulation of large lipid droplets and irregular shapes of mitochondrial cristae. However, serum cortisol and ACTH levels were lower in Aotus than in Saimiri [118].

Three zones of the adrenal cortex have been identified in Rhesus macaques. Expression of SULT2A1 and cytochrome b5 has been detected in the ZR. This indicates active androgen production in this region. Similar to humans, expression of 3β-HSD was not observed in this study [119, 120]. The enzyme 17α-hydroxylase is also present in the Rhesus macaques [121].

However, Patterson et al. found that newborn male marmosets have a fetal zone that produces androgens, similar to humans. In contrast, adult male marmosets do not appear to have an actively functioning androgen-producing ZR [121].

Despite the fact that the adrenal glands of primates and humans are highly similar, this model has limitations due to the conditions of confinement, the complexity of interacting with these animals, and the costs associated with conducting research.

#### Spiny mice

Mice of the genus *Acomys*, commonly known as spiny mice, are rodents that inhabit the deserts of the Middle East, Africa, and South Asia, known for their complex social organisation. They are named for the prominent spiky hairs on their backs [122]. Spiny mice belong to the Muridae family, which comprises almost a third of all rodent diversity [123]. Unlike other rodents, where organ development completes after birth, many internal organs in spiny mice develop during the embryonic period [124]. This has led some researchers to propose the use of spiny mice as models for embryogenesis [125]. Young spiny mice are highly mobile compared to neonates from other laboratory rodents, and they can consume solid food from the second day of life [126].

Spiny mice have been used as research animals since at least 1911, but interest in them intensified in 2012 when their regenerative capabilities were first discovered [126, 127]. Spiny mice were used as models for the study of diabetes, as they showed spontaneous Langerhans islet degeneration, a tendency toward hyperglycemia, and obesity-related diabetes, but without insulin resistance [128]. Furthermore, physiological studies were performed to assess kidney function, which revealed a high concentration of urea in the urine, possibly due to habitat [129]. Bellofiore et al. described spiny mice as the first rodents to exhibit a menstrual cycle that lasts 8–9 days [130].

The study of spiny mouse adrenal glands is relatively recent, but it is already known that they are more similar to human adrenal glands compared to the more usual laboratory mouse and rat models [10]. The adrenal glands of spiny mice are covered by a connective tissue capsule and contain the cortex and medulla. In the cortex, three sequential zones are distinguished: ZG, ZF, and ZR. Furthermore, immunohistochemical staining has shown the expression of 17α-hydroxylase, 3β-HSD, CYB5a in the cortex on day 30 of gestation, which is necessary for cortisol and androgen synthesis [131, 132]. In a study by Lamers W. H. et al., cortisol was found to be the primary glucocorticoid in spiny mice, while corticosterone is dominant in rats [125]. Another study (Quinn TA et al.) identified chromaffin cells in the medulla using antibodies to tyrosine hydroxylase, as well as presumptive sympathetic neurones detected by antibodies to synaptophysin and nonphosphorylated neurofilament H [131].

The advantages of this model include its similar structure to the human adrenal glands, both morphologically and functionally, as well as its simple laboratory maintenance requirements.

## Conclusions

The animals studied in this review could potentially serve as models for studying various human adrenal diseases. Each of them has its own characteristics and advantages, but also has certain limitations that may restrict their use in this role.

Among the species considered, spiny mice and golden hamster stand out for their morphological and functional similarity to the human adrenal glands. Their anatomical structure, including the arrangement of adrenal cortex zones and the expression of important enzymes for hormone synthesis, makes them the most promising model for research in the field of CAH.

Further molecular genetics and physiological studies of *Acomys* mice and golden hamsters may help establish their complete similarity to human adrenal glands and identify potential similarities and differences in pathologies. This could lead to the development of a “gold standard” animal model for studying human adrenal diseases, which in turn contributes to a more accurate understanding of the mechanisms of these diseases and the development of new treatment methods.

## Data Availability

Raw data used for the figure presented in this article will be made available upon request to the corresponding author.

## Abbreviations

17β-HSD: 17β-hydroxysteroid dehydrogenase
3β-HSD: 3β-hydroxysteroid dehydrogenase
ACTH: adrenocorticotropic hormone
CAH: congenital adrenal hyperplasia
CYT-B5: cytochrome B5 enzyme
DHEA: dehydroepiandrosterone
DHEA-S: dehydroepiandrosterone sulphate
IZ: intermediate zone
JZ: juxtamedullary zone
M: medulla
POR: P450 oxidoreductase
StAR: acute steroidogenic regulatory protein
SULT 2A1: sulfotransferase
ZF: zona fasciculata
ZG: zona glomerulosa
ZR: zona reticularis
